# Combination of Enzastaurin and Ibrutinib synergistically induces anti-tumor effects in diffuse large B cell lymphoma

**DOI:** 10.1186/s13046-019-1076-4

**Published:** 2019-02-18

**Authors:** Yizi He, Jiao Li, Ning Ding, Xiaogan Wang, Lijuan Deng, Yan Xie, Zhitao Ying, Weiping Liu, Lingyan Ping, Chen Zhang, Yuqin Song, Jun Zhu

**Affiliations:** Key laboratory of Carcinogenesis and Translational Research (Ministry of Education), Department of lymphoma, Peking University Cancer Hospital & Institute, 52 Fucheng Road, Haidian District, Beijing, 100142 People’s Republic of China

**Keywords:** Diffuse large B cell lymphoma, PKCβ inhibitor, BTK inhibitor, Targeted therapy, Drug combination

## Abstract

**Background:**

Diffuse large B cell lymphoma (DLBCL) is the most common form of lymphoma. Although durable remissions can be achieved in more than half of these patients, DLBCL remains a significant clinical challenge, with approximately 30% of patients not being cured. BCR-associated kinases (SYK, BTK, and PI3K) inhibitors have exhibited encouraging pre-clinical and clinical effects, as reported by many researchers. Early studies demonstrated that protein kinase C-β (PKCβ) inhibitors alter phosphorylation level the Bruton’s tyrosine kinase (BTK), which leads to enhanced BTK signaling. Here, for the first time, we investigate whether the combination of PKCβ inhibitor enzastaurin and BTK inhibitor ibrutinib has synergistic anti-tumor effects in DLBCL.

**Methods:**

In vitro cell proliferation was analyzed using Cell Titer-Glo Luminescent Cell Viability Assay. Induction of apoptosis and cell cycle arrest were measured by flow cytometry. Western Blotting analysis was used to detect the essential regulatory enzymes in related signaling pathways. RNA-seq was conducted to evaluate the whole transcriptome changes brought by co-treatment with low doses of enzastaurin and ibrutinib. The synergistic anti-tumor effects of enzastaurin and ibrutinib were also evaluated in vivo.

**Results:**

Combination of enzastaurin and ibrutinib produced a lasting synergistic effect on the survival and proliferation of DLBCL cells, including reduction of proliferation, promoting apoptosis, inducting G1 phase arrest, preventing cell invasion and migration, and down-regulating activation of downstream signaling. More importantly, whole-transcriptome changes results showed that combination therapy worked synergistically to regulate whole-transcriptome expression compared with enzastaurin and ibrutinib alone. Co-treatment with low doses of enzastaurin and ibrutinib could effectively downregulate BCR, NF-κB, JAK and MAPK related signaling pathway. Furthermore, the mRNA expression analysis further indicated that co-treatment significantly decreased the mRNA levels of NOTCH1. The combination effect in inhibiting proliferation of DLBCL cells probably was realized through suppression of NOTCH1 expression. Finally, the anti-tumor activity of co-treatment also was demonstrated in vivo.

**Conclusions:**

Combination of enzastaurin and ibrutinib had synergistic anti-tumor effects in DLBCL, independent of molecular subtype. These results provided a sound foundation for an attractive therapeutic treatment, and the simultaneous suppression of BTK and PKCβ might be a new treatment strategy for DLBCL.

**Electronic supplementary material:**

The online version of this article (10.1186/s13046-019-1076-4) contains supplementary material, which is available to authorized users.

## Background

Diffuse large B cell lymphoma (DLBCL), the most common form of lymphoma, is characterized by a heterogeneous tumor entity that can vary in morphologic, biological, immunophenotypic, and clinical presentation, as well as therapeutic outcome [1]. Gene expression profiling can be used to differentiate two subtypes of DLBCL, germinal center B-cell like (GCB) and activated B-cell-like (ABC) subgroups of DLBCL, leaving approximately 10~20% of cases “unclassified” [1]. ABC and GCB DLBCL are characterized by activation of different cellular pathways, posing a major barrier for developing a clear understanding of tumor development, maintenance, and response to therapy [2]. Although durable remissions are achieved in more than half of DLBCL patients, the disease remains a major clinical challenge, with approximately 30% of patients not being cured [3]. Especially as relapsed/refractory DLBCL patients involve poor survival, novel and effective therapeutic strategies are urgently needed.

Abnormal B-cell receptor (BCR) signaling has been implicated in the pathogenesis of B-cell malignancy, which is widely appreciated as one of the primary mechanisms underlying disease progression [4, 5]. Continuous activation of BCR in ABC-type DLBCL leads to the phosphorylation and activation of regulatory and adaptor proteins, such as spleen tyrosine kinase (SYK), Bruton’s tyrosine kinase (BTK), and protein kinase C-β (PKCβ), especially in ABC-type DLBCL [2, 6–8]. By contrast, oncogenic signaling in GCB DLBCL is typically initiated and reinforced by sharing a dependence on PI3K/mTOR signaling, which is independent of nuclear factor κB (NF-κB) [9, 10]. In recent years, an increasing number of studies have focused on the therapeutic inhibition of BCR signaling, especially combination-based therapeutic regimens for treating DLBCL [6, 11].

Enzastaurin, a potent and selective oral inhibitor of several PKC isoforms, has been shown to regulate the PI3K/AKT/mTOR, MAPK, and JAK/STAT pathways in solid and hematological malignancies [12–14]. Although enzastaurin showed promising result in preclinical studies and Phase I/II clinical trials in DLBCL, recent Phase III clinical trials did not meet the primary end point [15–17]. Interestingly, some researchers have found that PKCβ works as a feedback loop inhibitor of BTK activation, which modulates signaling pathways via altering BTK membrane localization [18, 19]. PKCβ downregulates BTK activation via both transphosphorylation at Tyr551 and autophosphorylation at Tyr223. Thus, enzastaurin-mediated inhibition of PKCβ leads to enhanced membrane targeting of BTK, increased phosphorylation of PLCγ2, and amplified BCR-mediated Ca^2+^ signaling [19].

Ibrutinib is an irreversible small molecule BTK inhibitor that has clearly demonstrated promising therapeutic effects in a variety of B cell malignancies [2, 20–22]. Therefore, we aimed to investigate whether the combination of PKCβ inhibitor enzastaurin and BTK inhibitor ibrutinib has synergistic anti-tumor effects in DLBCL. We demonstrated that low doses of enzastaurin and ibrutinib act synergistically to suppress growth of both ABC and GCB DLBCL cells in vitro and in vivo. These results provide support for future investigation of the combination of enzastaurin and ibrutinib as an attractive therapeutic option for patients with both subtypes of DLBCL.

## Methods

### Cell lines and cell culture

HBL-1, TMD8, OCI-LY7 cell lines were generously provided by Dr. Fu, University of Nebraska Medical Center (Omaha, NE, USA). SU-DHL-2 and SU-DHL-6 cells were obtained from American Type Culture Collection (Manassas, VA, USA). Cells were grown in RPMI 1640 medium (Gibco, Life Technologies, CA, USA) supplemented with 10–20% fetal bovine serum (Gibco, Life Technology, CA, USA), penicillin/ streptomycin, glutamine, beta-mercaptoethanol. Except for OCI-LY7, which was maintained in IMDM (Gibco, Life Technology, CA, USA) supplemented with beta-mercaptoethanol, penicillin/ streptomycin, and 20% heparinized human plasma. All cell lines were maintained in a humidified 5% CO_2_ incubator at 37 °C. Identification of all DLBCL cell lines was confirmed by short tandem repeat DNA fingerprinting analysis (Applied Biosystems, Foster City, CA, USA).

### Drugs and reagents

Enzastaurin was a gift from Denovo Biopharma (San Diego, USA), and ibrutinib was purchased from Medchem Express (NJ, USA). It was initially dissolved in 100% DMSO (Sigma–Aldrich, Darmstadt, Germany) at a concentration of 10 μM and stored in − 80 °C. Primary and secondary antibodies were listed in additional file (Additional file 1: Table S1).

### Analysis of cell proliferation

Cells were seeded in a 96-well culture plate at a density of 3000 cells per 100 μl and treated with different concentrations of enzastaurin and ibrutinib for 72 h. Cells were counted and viability was assessed using Cell Titer-Glo Luminescent Cell viability assay system (Promega, Madison, WI, USA). Luminescent signals were measured by LMax II (Molecular Devices, Sunnyvale, CA, USA). Inhibition rates were calculated following the formula: inhibition rates = (1- dosing/control) × 100%.

### Apoptotic cells and cell-cycle assays

Cells were treated with vehicle or indicated concentrations of enzastaurin and ibrutinib for 48 h for apoptosis and cell cycle analysis. For apoptosis assays, cells were stained with annexin V-APC (Biolegend, CA, USA) according to the protocol. For cell cycle assays, cells were stained with PI staining buffer (Sigma–Aldrich, Darmstadt, Germany) according to the manufacturer’s protocol. Finally, the labeled cells were analyzed using BD Accuri C6 flow cytometer (BD, Biosciences, San Jose, CA).

### Real-time reverse transcription-PCR (qRT-PCR) assay

Total cellular RNA was extracted using Trizol reagent (Life Technologies, Carlsbad, CA) and cDNA was synthesized using TransScript First-Strand cDNA Synthesis SuperMix (TransGen Biotech, Beijing, China). qRT-PCR analysis was performed using Go Taq qPCR Master Mix (Promega Corporation, Madison, USA). Specific primers for NOTCH1 (Forward: 5′–TCCACCAGTTTGAATGGTCAAT-3′; Reverse: 5′-CGCAGAGGGTTGTATTGGTTC-3′) and GAPDH (Forward: 5′-GCACCGTCAAGGCTGAGAAC-3′; Reverse: 5′-TGGTGAAGACGCCAGTGGA-3′) were used to perform qRT-PCR. All reactions were run in Applied Biosystems 7500 Fast Real-Time PCR System (Applied Biosystems, Woburn, MA, USA), mRNA expression data were calculated using the following equation: RQ = 2^-ΔΔCt^.

### Western blotting and signaling assays

Harvested cultured cells were lysed in RIPA buffer (Cell Signaling Technology, Danvers, MA) with protease/phosphatase inhibitor (Roche, Mannheim, Germany). Signaling proteins were detected by western blot as previously described [23]. Immunopositive bands were visualized using chemiluminescence detection system (Alpha Innotech, San Leandro, CA, USA) according to the manufacturer’s instructions.

### Invasion and migration assay

Cells were treated with vehicle or indicated concentrations of enzastaurin and ibrutinib for indicated time in FBS-free RPMI 1640. For cell invasion assays, cells were placed into Matrigel basement membrane matrix-coated upper chambers in a transwell plate with 8.0 μM pores (Corning Costar, NY, USA). For cell migration assays, cells were seed into transwell with 8.0 μm pore polycarbonate membrane insert (Corning Costar, NY, USA). The lower portion of the chamber contained 30% FBS for use as a chemoattractant. After 24 h (48 h), the number of cells migrating (invading) into the lower chamber were counted using Cell Titer-Glo Assays. Invasive and migration abilities were determined by the number of viable cells in the lower chamber.

### Gene expression profiling and Kyoto encyclopedia of genes and genomes (KEGG) pathway enrichment analysis

Cells were treated with the indicated drug alone or in combination for 24 h, and then total RNA was isolated. Total RNA (3 μg) was converted to cDNA using TransScript First-Strand cDNA Synthesis SuperMix. RNA quantification and qualification, library preparation, clustering and sequencing, read mapping and data processing were performed in Novogene Bioscience (Beijing, China). Differential expression analysis of two groups (two biological replicates per group) was performed using the DESeq2 R package (1.16.1). Corrected *P*-value of 0.05 and absolute foldchange of 2 were set as the threshold for significantly differential expression. To analyze the underlying mechanism of the sets of genes which were differentially expressed following each treatment, we used clusterProfiler R package to test the statistical enrichment of differential expression genes in Kyoto Encyclopedia of Genes and Genomes (KEGG) pathways.

### Lentivirus packing and infection

Lentiviral vectors (GV493) containing green fluorescent protein (GFP) (shControl) or NOTCH1-specific short hairpin RNA (shNOTCH1, sequence #1: 5′-TGCCAACATCCAGGACAACAT-3′) were constructed, packed, and purified by Genechem (Shanghai, China). Cells were infected with shControl, shNOTCH1, at MOI 1: 100 and cultured for > 72 h to be used for the downstream experiments. The depletion efficiency was assessed by western blot analysis.

### Detection of treatment efficacy in vivo

All animal experiments were performed in compliance with the Guide for the Care and Use of Laboratory Animals and in accordance with the ethical guidelines of CrownBio (Beijing, China). Female immune-deficient NPG mice (NOD-Prkdcscid Il2rgnull), six to eight weeks old, were obtained from HFK Bioscience Co.Ltd. (Beijing, China). HBL-1 tumor cells (5 × 10^6^) in serum-free medium with matrigel (1:1 ratio) were injected subcutaneously into the area under the right flank of each mouse. When the tumor reached 100–150 mm^3^, mice were randomly divided into four groups (control, treated with enzastaurin, treated with ibrutinib, treated with both enzastaurin and ibrutinib). Enzastaurin (125 mg/kg, dissolved in 10% Acacia) was administered twice daily orally and/or ibrutinib (12 mg/kg, dissolved in 1% methylcellulose, 0.4% Cremophor® EL) was administered once daily orally for 21 days. Tumor volume (V) and body weight were monitored two to three times per week. The tumor volume (V) was calculated as V = (length×width^2^) /2. Tumor tissue samples were collected from all groups at 4 h after the last dose.

### In situ apoptosis quantification by TUNEL

TUNEL is a method for detecting DNA fragmentation by labeling the 3′- hydroxyl termini in the double-strand DNA breaks generated during apoptosis. HBL-1 tumor samples were fixed in 4% paraformaldehyde, embedded in paraffin and cut into 5 μm sections. A TUNEL assay was then conducted to examine DNA fragmentation using an in situ cell death detection kit (Cat No. 11684795910, Roche, Mannheim, Germany) according to the manufacturer’s instructions.

### Immunohistochemistry

Immunohistochemistry stains for Ki-67, p-BTK and p-PKCβ were performed in the department of pathology of Peking University Cancer Hospital using the standard streptavidin-biotin-peroxidase immunostaining procedure. The slides were incubated with primary antibody overnight at 4 °C and then with HRP-conjugated secondary antibody at room temperature for 30 min. DAB was used for staining. The intensity and density of the staining were examined in a double-blinded manner by two independent pathologists from the department of pathology in Peking University Cancer Hospital & Institute. Primary antibodies were listed in Additional file 1: Table S1.

### Statistical analysis

All experiments in vitro were independently done more than three times. The SPSS 22.0 statistical software (IBM, New York, NY, USA) was used for all analyses. Data were analyzed using paired or unpaired Student’s t test comparisons or one-way ANOVA. *P* values <0.05 were accepted as statistically significant. The combination index (CI) for drug combination was determined according to the Chou-Talalay method using the CalcuSyn software (version 2, Biosoft, Cambridge, UK). CI values <1, =1, and > 1 indicates synergism effects, additive effects, and antagonism effects, respectively.

## Results

### Enzastaurin inhibited proliferation of ABC and GCB cell lines in a dose-dependent manner and upregulates BTK phosphorylation

**Fig. 1:**
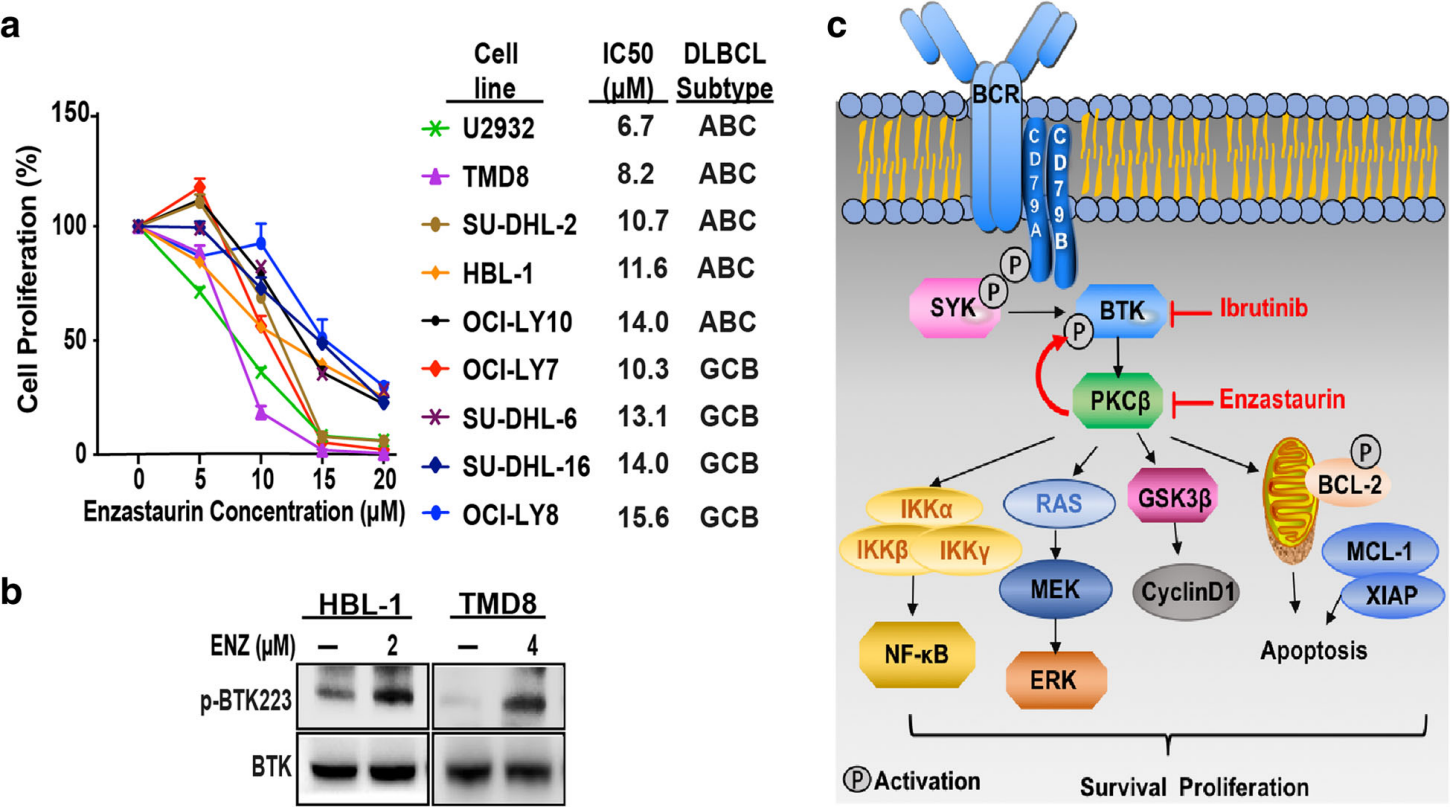
Enzastaurin inhibited proliferation of ABC and GCB cell lines and up-regulated phosphorylation of BTK. **a** ABC (HBL-1, TMD8, U2932, SU-DHL-2, OCL-LY10) and GCB (SU-DHL-6, SU-DHL-16, OCI-LY7, OCI-LY8) lymphoma cell lines were cultured with DMSO or enzastaurin with increasing doses up to 20 μM for 72 h. The cell viability was measured by Cell Titer-Glo luminescent cell viability assay. Each cell line was analyzed in triplicate, and data are shown as mean ± SD. **b** Western blot analysis of p-BTK levels in HBL-1and TMD8 cells after DMSO or enzastaurin treatment for 2 h. **c** BCR signaling representation. Enzastaurin and ibrutinib block some effectors downstream of the BCR

To determine the effect of enzastaurin on the survival of DLBCL cell lines, we cultured nine cell lines in the presence of enzastaurin (0 to 20.0 μM) for 72 h. As shown in Fig. 1a, treatment with enzastaurin resulted in a dose-dependent inhibition of cell proliferation, with a 50% inhibitory concentration (IC50) values ranging between 6.7 and 15.6 μM (Fig. 1a). We confirmed that treatment with enzastaurin effectively reduced the viability of DLBCL cells, and there was no statistical difference between ABC and GCB cells lines (*p* = 0.48).

PKCβ is a common signaling target that lies downstream of BTK. Surprisingly, we observed that HBL-1 and TMD8 cells exhibited notable upregulation of phosphorylated BTK (p-BTK) upon treatment with enzastaurin (Fig. 1b). These results suggest that although inhibition of PKCβ is therapeutically effective in DLBCL cells, it also leads to positive regulation of BCR signal pathway. Thus, while pharmacological inhibition of enzastaurin attenuated some branches of BCR signaling pathways, inactivation of these pathways can be compensated by upregulation of other pathways (Fig. 1c). These compensatory pathways greatly limit the effectiveness of enzastaurin in DLBCL, especially as a monotherapy.

### Synergistic effects of enzastaurin and ibrutinib on the induction of cell death in DLBCL cell lines

**Fig. 2:**
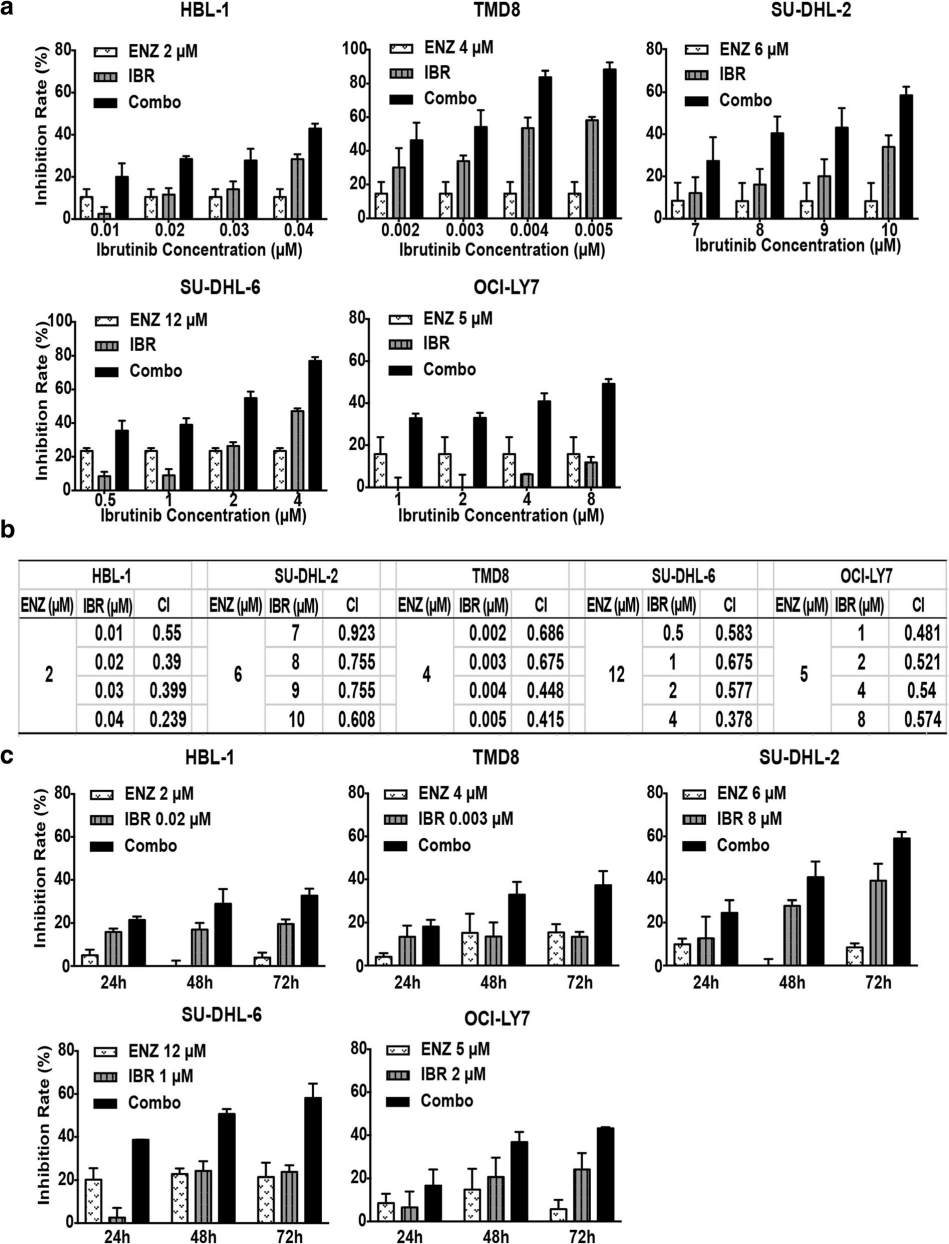
Synergistic effects of enzastaurin and ibrutinib on the induction of cell death in DLBCL cells. **a** Cell viability of HBL-1, TMD8, SU-DHL-2, OCI-LY7 and SU-DHL-6 incubated with varying concentrations of ibrutinib, in the presence or absence of fixed minimally toxic concentrations of enzastaurin for 72 h. Cell Titer-Glo luminescent assay were performed to detect the inhibition rate. **b** Combination index form for each combination of enzastaurin and ibrutinib was calculated by CalcuSyn software. CI values less than 1.0 denote a synergistic interaction. **c** Cells were treated with indicated concentration of enzastaurin and ibrutinib for different time intervals (24 h, 48 h, 72 h), cell viability was monitored by Cell Titer-Glo luminescent assay. Inhibition rates = (1-dosing/control) × 100%. Error bars represent mean ± SD of triplicates. ENZ, enzastaurin; IBR, ibrutinib; Combo, enzastaurin combined with ibrutinib

Our initial results suggested that simultaneous inhibition of PKCβ and BTK would block BCR signaling and induce cell death in DLBCL cells. Based on the cytotoxicity of enzastaurin and ibrutinib, we exposed the GCB (SU-DHL-6 and OCI-LY7) and ABC (HBL-1, TMD8 and SU-DHL-2) lymphoma cells to minimally toxic concentration of enzastaurin, together with increasing concentrations of ibrutinib in combination for 72 h. The toxicity of each treatment was assessed by measuring the rate of growth inhibition. Notably, DLBCL cells (SU-DHL-2 and SU-DHL-6) that responded poorly to enzastaurin or ibrutinib as a single-agent therapy were exquisitely sensitive to combination treatment with these two drugs (Fig. 2a). Combination therapy with enzastaurin and ibrutinib greatly increased the inhibition rate of DLBCL cell growth irrespective of the molecular subtype or the level of responsiveness to ibrutinib monotherapy (Fig. 2a).

To further confirm the synergistic effect of enzastaurin and ibrutinib in DLBCL, CI values were calculated (Fig. 2b). The combined therapy showed a strong synergistic inhibitory effect on the growth of HBL-1, TMD8, SU-DHL-2, SU-DHL-6 and OCI-LY7 cells at all tested doses, with CI value ranging from 0.239 to 0.686. The synergistic effects in SU-DHL-2 were weak, with a CI range of 0.608–0.923. Overall, the combinations of enzastaurin and ibrutinib thus exhibited synergistic effects in GCB and ABC subtypes of DLBCL cell lines at all doses examined (CI < 1, Fig. 2b).

Time-course analysis of cell death further indicated that that prolonged exposure to the combination had an even greater effect on inhibition of cell proliferation (Fig. 2c). Thus, the combination of enzastaurin and ibrutinib demonstrated long-term synergistic effects on the survival and proliferation of DLBCL cells, independent of their subtype.

### The combination of enzastaurin and ibrutinib promoted apoptosis and induced G1 arrest in DLBCL cells

**Fig. 3:**
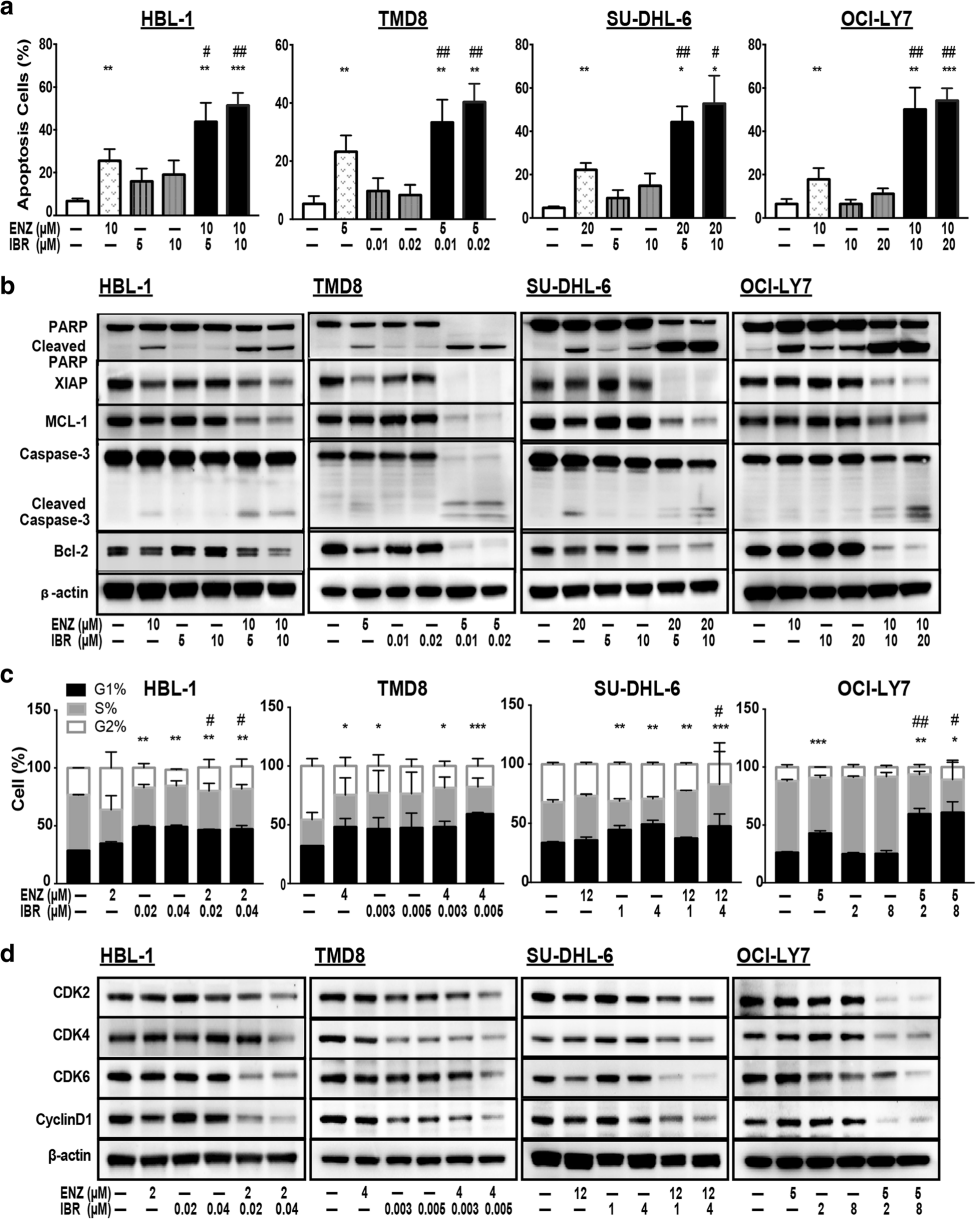
The combination of enzastaurin and ibrutinib promoted apoptosis and induced G1 phase arrest. **a** Combination treatment prompted apoptosis in DLBCL cells. Cells pre-treated with indicated concentrations of ibrutinib in the presence or absence of fixed concentration of enzastaurin for 48 h were stained with annexin V-APC, then apoptosis was assessed using flow cytometry. Apoptosis cells were determined by APC^+^ cells. **b** The combination treatment mediated expression of proteins associated with apoptosis in DLBCL cells. After 48 h of exposure to enzastaurin and/or ibrutinib in combination, proteins were extracted from cells of different groups and proteins associated with apoptosis were analyzed by western blot. **c** Co-treatment with enzastaurin and ibrutinib induced G1 phase arrest in DLBCL cells. Cells were treated with different concentrations of ibrutinib in the presence or absence of fixed concentration of for enzastaurin 48 h, and then stained with propidium iodide (PI). Cell cycle was assessed using flow cytometry. **d** The combination treatment group mediated alterations in proteins associated with G1/S transition in DLBCL cells. After 48 h of exposure to indicated concentration of enzastaurin and ibrutinib in combination, proteins were extracted from cells of different groups and proteins were analyzed by western blot. Error bars represent the result and SD of three different experiments. * *p* < 0.05, ** *p* < 0.01, *** *p* < 0.001, compared with control group; # *p* < 0.05, ## *p* < 0.01 compared with enzastaurin group

To determine whether inhibition of cell growth by co-treatment with enzastaurin and ibrutinib was associated with apoptosis and/or cell cycle arrest, we analyzed levels of apoptosis in four cells lines after 48 h exposure to the indicated concentrations of enzastaurin and/or ibrutinib. In HBL-1, the combination of enzastaurin with two different doses of ibrutinib induced 43.8 ± 8.7% or 51.4 ± 5.9% apoptosis respectively, as measured by annexin V staining; these values were greater than those cells treated with each single agent alone (enzastaurin = 25.5 ± 5.4%, ibrutinib = 15.9 ± 6.0% and 19.0 ± 6.7%, Fig. 3a). Thus, co-treatment with enzastaurin and ibrutinib has a synergistic effect on promoting apoptosis. Consistent with the results of annexin V staining, expression of proteins associated with apoptosis also changed in response to co-treatment in HBL-1 cells (Fig. 3b). Treatment with either enzastaurin or ibrutinib slightly increased expression the active forms of poly-ADP ribose polymerase (PARP) and caspase-3, but co-treatment dramatically increased these effects (Fig. 3b). Treatment with the combination also induced a sharp decrease in the expression of anti-apoptotic Bcl-2 family members, including Mcl-1, XIAP, and Bcl-2. Similar results were observed in TMD8, SU-DHL-6 and OCI-LY7 cells (Fig. 3a, b). Taken together, these results show that the con-administration of enzastaurin and ibrutinib promotes apoptosis through activation of the caspase-dependent and mitochondrial pathway in DLBCL cells, ultimately resulting in cytotoxicity.

In order to assess the effects of enzastaurin and ibrutinib on the cell cycle, we used flow cytometry to analyze the cell cycle profiles of treated cells (Fig. 3c). The percentage of HBL-1 cells in G1 phase increased from 28.5 ± 0.05% in the control group to 46.4 ± 0.84% and 47.2 ± 3.12% in the combination treatment groups. A corresponding decrease of cells in S phase also occurred. Similar results were observed in TMD8, SU-DHL-6 and OCI-LY7 cells (Fig. 3c). Consistent with these results, expression of CDK2, CDK4, CDK6 and Cyclin D1 substantially decreased in cells co-treated with enzastaurin and ibrutinib, whereas treatment with single agents only mildly affected the expression of these proteins known to play essential roles in the G1/S transition. Similar trend were observed in the other three cell lines (Fig. 3d). These data demonstrate that the combinations of enzastaurin and ibrutinib induced G1 phase arrest and the combination therapy suppressed cell proliferation by inducing both cell cycle arrest and initiation of apoptotic.

### Treatment with low doses of enzastaurin and ibrutinib synergistically inhibits migration and invasion in DLBCL

**Fig. 4:**
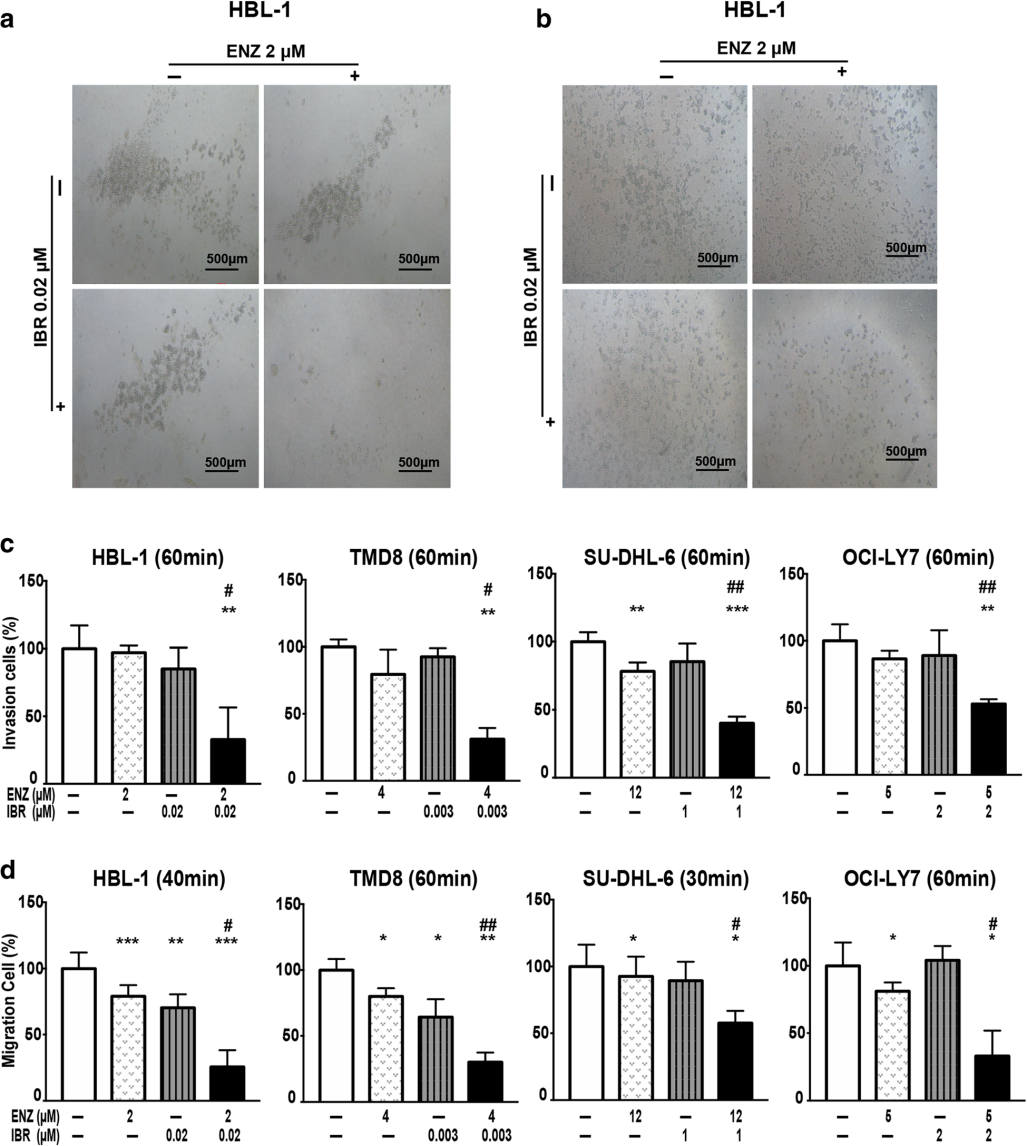
Treatment with enzastaurin and ibrutinib synergistically inhibits migration and invasion in DLBCL. (**a**-**b**) HBL-1 cells pre-treated with 2 μM enzastaurin and/or 0.02 μM ibrutinib for 60 min (40 min) were subjected to the invasion (migration) assay using Corning chamber. Representative images of cell invaded (**a**) and migrated (**b**) to the lower chamber are shown. (**c**-**d**) Co-treatment therapy suppressed invasion and migration of HBL-1, TMD8, OCI-LY7 and SU-DHL-6 cells. Cells pre-treated with indicated concentrations of enzastaurin and/or ibrutinib for different time were placed on transwell to determine cell migration and on transwell with pre-coated Matrigel to determine cell invasion. After 48 h (24 h) of incubation, the cells that invaded (migrated) to the lower chamber were counted. The mean percentage of cells invaded (**c**) and migrated (**d**) are shown. Results are expressed as percentages of controls in mean ± SD, data are representative of three independent experiments. * *p* < 0.05, ** *p* < 0.01, *** *p* < 0.001 compared with control group; # *p* < 0.05, ## *p* < 0.01 compared with enzastaurin group

In order to assess the possible effects of treatment with low doses of enzastaurin and ibrutinib on cell motility, we performed cell migration and invasion assays using DLBCL cells. For invasive abilities, treatment with enzastaurin or ibrutinib alone slightly suppressed invasive of HBL-1 cells, with 97.0 and 85.0% cells exhibiting invasion, respectively. In contrast, invasion was notably suppressed in cells treated with the combination of enzastaurin and ibrutinib, with only 32.8% of cells invading relative to the control group (Fig. 4a).

Analysis of migration revealed that treatment with the single agent reduced migration to 79.0 and 70.2% of HBL-1 cells, respectively. In contrast, the number of co-treated cells passing through the membrane was only approximately 25.5% of the control cells (Fig. 4b). Similar trends were observed in TMD8, SU-DHL-6 and OCI-LY7 cells, and detailed results are shown in the invasion and migration histogram (Fig. 4c). These findings demonstrate that enzastaurin and ibrutinib synergistically decrease cell migration and invasion, which are essential for DLBCL cell motility.

### Co-demonstration of enzastaurin and ibrutinib synergistically inhibit downstream signaling pathways

**Fig. 5:**
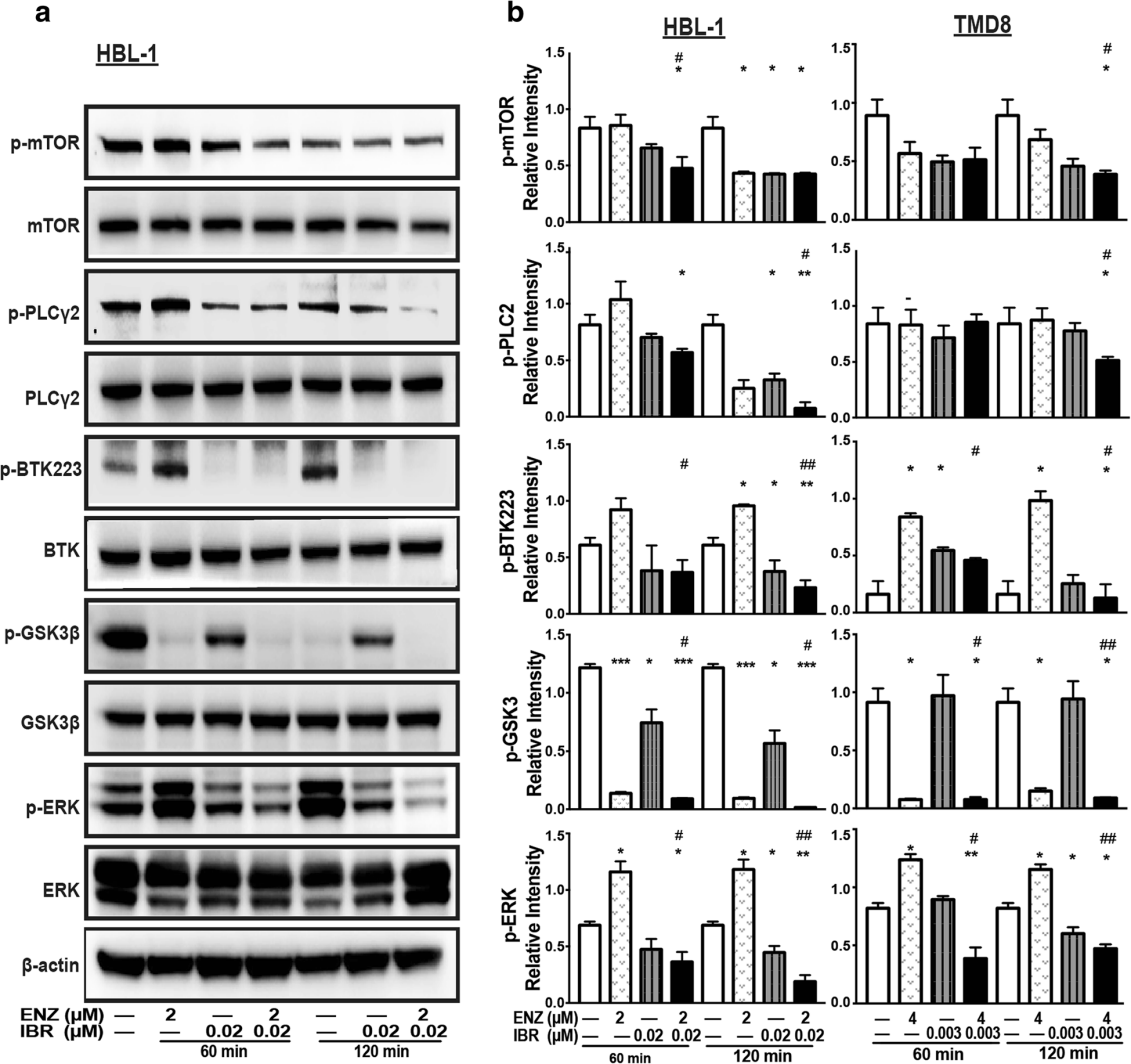
Co-demonstration of enzastaurin and ibrutinib synergistically inhibit downstream signaling pathways. **a** Cells were treated with indicated concentration of enzastaurin alone, ibrutinib alone or enzastaurin plus ibrutinib for 60 min and 120 min, and cells harvested for western blot analysis. **b** The relative phosphorylation levels of signaling mediators were quantified by measuring the relative intensity of phosphorylated bands to the corresponding total bands, results are presented as mean ± SD of three scans. * *p* < 0.05, ** *p* < 0.01, *** *p* < 0.001 compared with control group; # *p* < 0.05, ## *p* < 0.01 compared with enzastaurin group

To gain insight into the mechanism underlying the anti-proliferative effects of co-treatment with enzastaurin and ibrutinib in DLBCL models, we next investigated the changes of signal transduction pathways in treated cells. As shown in Fig. 5a, HBL-1 cells treated with low doses of enzastaurin monotherapy for 60 min and 120 min showed clearly reduced the phosphorylation of glycogen synthase kinase 3β (GSK3β), which serves as a biomarker for enzastaurin activity. Short-term and low-dose enzastaurin treatments had not significantly affect on the PKCβ phosphorylation (data not show), and increased expression of p-BTK, p-ERK. Similarly, treatment with Ibrutinib alone reduced levels of BTK phosphorylation, which was accompanied by a mild effect on phosphorylation of mTOR, PLCγ2, and ERK. However, co-treatment with enzastaurin and ibrutinib resulted in a greater reduction in phosphorylation of ERK, mTOR, PLCγ2, compared to each monotherapy alone (Fig. 5a, b). These results were also confirmed in TMD8 and SU-DHL-6 cells (Fig. 5b, Additional file 2: Figure S1). Overall, in contrast to single treatment, the combination of enzastaurin and ibrutinib appears to more effective inhibit signal transduction in both ABC and GCB cell models, indicating that co-treatment successfully suppress multiple signaling pathways downstream of BCR.

### Whole-transcriptome changes in DLBCL occur in response to the combination of enzastaurin and ibrutinib

**Fig. 6:**
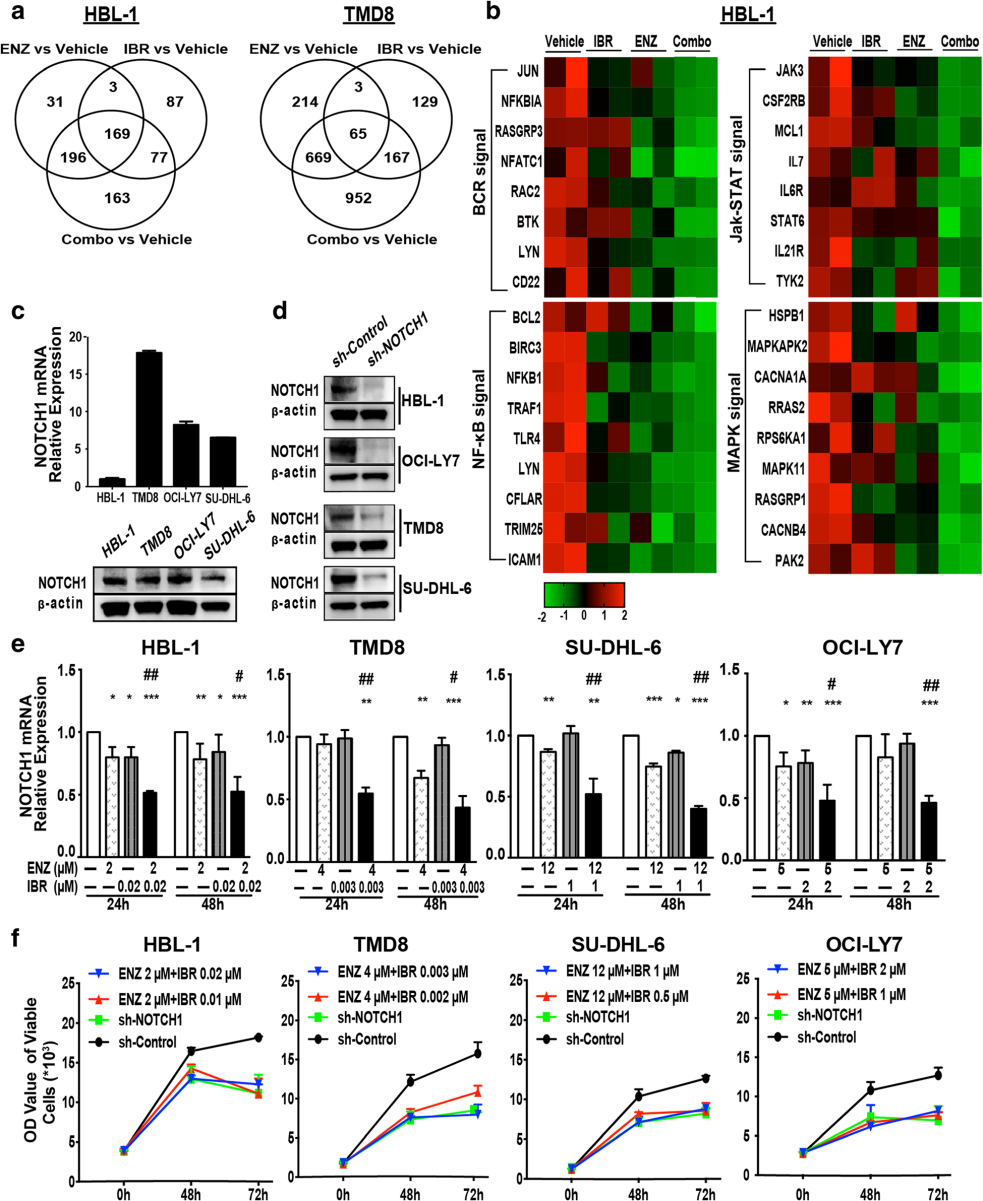
Whole-transcriptome changes in DLBCL occur in response to the combination of enzastaurin and ibrutinib. HBL-1 and TMD8 cells were exposed for 24 h with enzastaurin (HBL-1 2 μM, TMD8 4 μM) and/or ibrutinib (HBL-1 0.02 μM, TMD8 0.003 μM). RNA was collected for RNA sequencing. **a** Venn diagram illustrating the number of overlapping downregulated gene between different groups. **b** Significantly down-regulated genes from top ranked pathways (by KEGG) are represented in the heatmap. Colors scale bar represents from higher (red) to lower (blue) expression. Gene expression levels are expressed in FPKM values, differences shown in color scale after Z-score transformation. Down-regulated genes were determined by log2foldchange<0. FPKM, fragments per kilo base of exon per million fragments mapped. **c** The expressions of NOTCH1 in DLBCL cell lines were detected using qRT-PCR and western blot. **d** NOTCH1 knockdown by shRNA was validated by western blot in HBL-1, TMD8, OCI-LY7, SU-DHL-6 cells. β-actin is shown as a loading control. **e** The expressions of NOTCH1 gene were further confirmed in DLBCL cells after pre-treated with enzastaurin and/or ibrutinib. **f** The DLBCL cells were transfected with shRNA targeting NOTCH1, or treated with enzastaurin and ibrutinib for 48 h, 72 h. The cell viability of tumor cells was determined using the Cell Titer-Glo luminescent cell viability assay. Results are expressed as mean ± SD, data are representative of three independent experiments. * *p* < 0.05, ** *p* < 0.01, *** *p* < 0.001 compared with control group; # *p* < 0.05, ## *p* < 0.01 compared with enzastaurin group

In order to better understand the effects of combination therapy with low doses of enzastaurin and ibrutinib in DLBCL cells, we assayed whole-transcriptome changes by RNA-sequencing. Several hundred transcripts observed to be either up or down regulated by different treatments. Because the upregulated genes were not closely associated with these inhibitors, only the downregulated genes were further analyzed. Venn diagram was used to depict the number of downregulated genes associated with the different treatments (< 2 fold, *p* < 0.05). Enzastaurin and ibrutinib were less efficient as single agents, with 399 and 336 transcripts significantly downregulated, respectively, compared with 605 downregulated transcripts for the combination treatment. Approximately 91% of transcripts (365 genes) repressed by enzastaurin and 73% of transcripts (246 genes) repressed by ibrutinib were included in the combination group. Additionally, the combination treatment efficiently downregulated an additional 163 transcripts that had not been downregulated by either drug alone. Similar results were observed in TMD8 cells (Fig. 6a). Thus, co-treatment with enzastaurin and ibrutinib resulted in the downregulation of a broader set of genes compared to the treatment with either of the compounds alone.

Further analysis of downregulated genes showed that compared with vehicle treatment control, significantly downregulated genes from top ranked pathways (by KEGG) are represented in the heat map (Fig. 6b). Co-treatment with low doses of enzastaurin and ibrutinib effectively downregulated genes associated with BCR, NF-κB, JAK-STAT and MAPK signaling pathways. These pathway analysis results were also confirmed in TMD8 cells (Additional file 2: Figure S2), which consistent with those from Western blot results (Figs. 3, 5). Thus, combination therapy appeared to synergistically regulate whole-transcriptome changes.

To further assess the synergistic anti-tumor effects of enzastaurin and ibrutinib, we analyzed the expression of transcripts downregulated by the combination treatment using qRT-PCR. Compared with enzastaurin and ibrutinib monotherapy, combination treatment was able to decrease the mRNA expression of NOTCH1 more significantly (Fig. 6e). A strong body of evidence underscores the important oncogenic role of NOTCH1 in promoting changes in cellular metabolism, cell growth and proliferation, and enhanced the activity of signaling pathways [23–26]. Furthermore, aberrant NOTCH1 activity has emerged as an important oncogenic regulator of hematological malignancy [26]. The NOTCH1 mRNA and protein were expressed at medium-to-high levels in DLBCL cells (Fig. 6c). Thus, the anti-proliferative effects of the combination of enzastaurin and ibrutinib in DLBCL cells are likely due to suppression of NOTCH1 expression.

To validate the role of NOTCH1 downregulation in DLBCL cell survival and proliferation, we used shRNA transfection to knock-down NOTCH1expression (Fig. 6d). Silencing of NOTCH1 in DLBCL cells had anti-proliferative effects, indicating that NOTCH1 expression is important for the survival of DLBCL cells. Similar proliferation effects and timing were observed in NOTCH1 shRNA treatment and co-treatment with enzastaurin and ibrutinib, suggesting that the synergistic effects of the combination treatment may occur through downregulating NOTCH1 expression (Fig. 6f).

### Enzastaurin and ibrutinib have synergistic antitumor effects in a DLBCL models in vivo

**Fig. 7:**
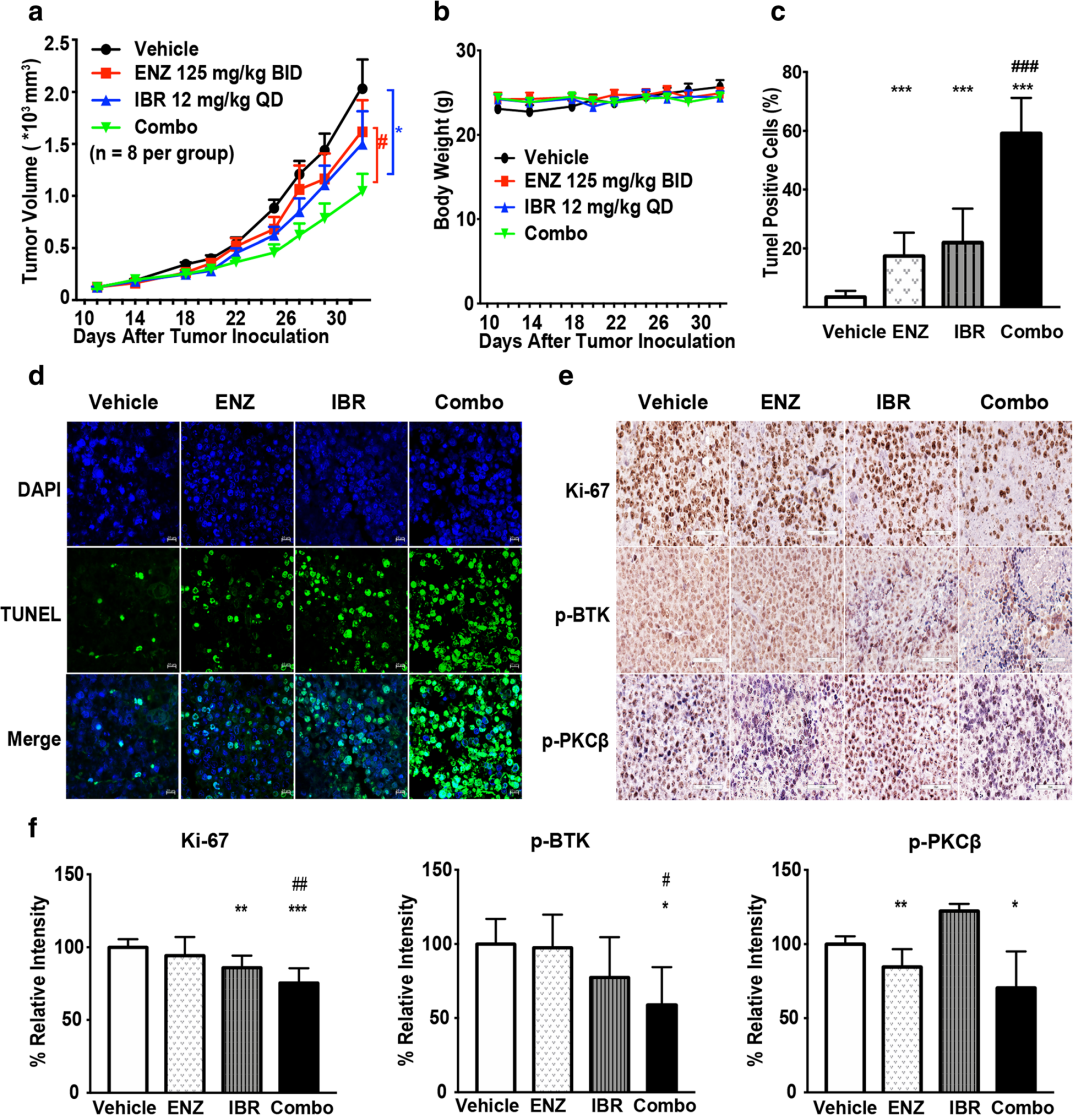
Enzastaurin and ibrutinib have synergistic antitumor effects in a DLBCL models in vivo. NPG mice subcutaneously inoculated with HBL-1 (5 × 10^6^) cells were randomized in four groups, respectively, treated as follow: enzastaurin (orally, 125 mg/kg, BID), ibtutinib (orally, 12 mg/kg, BID), combination of enzastaurin plus ibtutinib and control vehicle (*n* = 8 per group). **a** Tumor size curves derived from HBL-1 xenograft mouse model. **b** Body weight curves derived from HBL-1 xenograft mouse model. **c**-**d** Apoptosis of tumor tissue was assessed by the TUNEL assay; the nuclei were counterstained with DAPI. Representative images show apoptotic/fragmented DNA (green staining) and the corresponding cell nuclei (blue) staining. Scale bar 10 μM. Results are expressed as mean ± SEM. **e**-**f** Immunohistochemistry assay of Ki-67, p-BTK and p-PKCβ protein expression in xenograft tumors. **e** Representative pictures of immunohistochemistry staining in sections are shown (Scale bar 60 μM). **f** The data represents the density of positivity cells for each section. Values are expressed as percentages of vehicle in mean ± SD, * *p* < 0.05 compared with vehicle group, ** *p* < 0.01 compared with vehicle group, *** *p* < 0.001 compared with control group; # *p* < 0.05 compared with enzastaurin group, ## *p* < 0.01 compared with enzastaurin group

Finally, we assessed the ability of enzastaurin, alone and in combination with ibrutinib, to reduce tumor growth in a lymphoma model, in which ABC-DLBCL HBL-1 cells were engrafted in NPG mice (Fig. 7). Enzastaurin or ibrutinib monotherapy resulted in a smaller reduction in tumor volume relative to the control. Compared with control and monotherapy, tumor volumes were significantly smaller in mice treated with the combination treatment (*p* < 0.05, Fig. 7a). Treatment was well tolerated, and no mice lost weight obviously or died (Fig. 7b). At the end of the experiment, neither enzastaurin (811.28 ± 182.10 mg) nor ibrutinib (719.25 ± 156.71 mg) significantly inhibited tumor growth compared with that of the vehicle group (1075.29 ± 152.56 mg), while the co-treatment robustly suppressed tumor growth and restrained tumor weight (444.65 ± 87.64 mg, Additional file 2: Figure S3). To further evaluate the apoptosis, proliferation, and BCR signal status of tumor tissue post different treatments, TUNEL, Ki-67, p-BTK and p-PKCβ was investigated and quantified in paraffin sections of tumor samples collected from HBL-1 xenografts. As shown in Fig. 7c and d, combination therapy of enzastaurin and ibrutinib induced a notable increase of apoptosis compared with each agent alone. Moreover, co-treatment with enzastaurin and ibrutinib produced a more significant decrease of Ki- 67, p-PKCβ and p-BTK expression than the monotherapy achieved (Fig. 7e, f). Thus, these results demonstrate that the co-treatment of enzastaurin with ibrutinib has synergistic activity in preclinical models, confirming our in vitro findings.

## Discussion

DLBCL is a heterogeneous lymphoma, and although the introduction of rituximab has greatly improved clinical outcomes, it still proves incurable in 30%~ 40% of all cases [27]. One of the most important reasons underlying negative outcomes is that ABC and GCB DLBCLs exhibit activation of different signaling pathways. The ABC subtype is characterized by mutations in MYD88, CARD11, CD79A and CD79B, and constitutive activation of NF-κB signaling, features associated with less favorable clinical outcome [6, 7]. In contrast, GCB subtype is more frequently characterized by activation of the PI3K/AKT pathway, rather than NF-κB pathway [10]. These differences in signaling translate into different levels of tumor aggressiveness and differential response to therapy [28]. The crucial role played by the BCR signaling pathways in DLBCL has prompted the development of targeted kinase inhibitors, including inhibitors of BTK, PI3K, SYK and PKCβ, representing promising potential therapeutic strategies for DLBCL patients [29]. Here, for the first time, we demonstrate that combination treatment with enzastaurin and ibrutinib augments anti-tumor effects of the single agents in DLBCL in vitro and in vivo. These effects may be due to inactivation of related signaling pathways and downregulation NOTCH1 expression.

Enzastaurin is a relatively well-studied anti-tumor agent. Preclinical evaluation of enzastaurin has shown promising results in cutaneous T-cell lymphoma, B-cell lymphoma, multiple myeloma (MM), Waldenstrom’s macroglobulinemia (WM) and other solid tumors [30–32]. With respect to DLBCL, 22% of DLBCL tumor samples have been found to be positive for PKCβ expression, defined as immunostaining of > 50% of cells [33]. Furthermore, PKCβ expression is a useful marker of poor prognosis in DLBCL. Phase I/II studies of enzastaurin have shown that it is well tolerated in DLBCL patients, 15% (8/55) of the patients experienced prolonged freedom from progression (FFP ≥ 4 cycles) and 7% (4/55) of patients experienced FFP 20~50 months [16, 17]. However, similar results have not be observed in a phase III clinical trial (PRELUDE), which showed that enzastaurin monotherapy did not significantly improve disease-free survival (DFS) in high-risk DLBCL patients after remission of B cell lymphoma. These results have essentially halted the development of enzastaurin as a monotherapy in DLBCL. A large phase 3 global clinical trial was launched to assess enzastaurin plus R-CHOP in DLBCL patients with the genomic biomarker DGM1, identifying a novel genetic biomarker related to drug efficacy, which could improve the efficacy and outcomes of enzastaurin.

Analysis of failed therapeutics presents an opportunity for improvement through both preclinical and clinical investigations of therapeutic combinations. Prior studied have noted the combination treatment with HDACi and enzastaurin exhibit a synergistic effect in DLBCL. HDACi may increase the expression of PKCβ, leading to activation of survival signals [14]. Additionally, therapeutic regimens composed of enzastaurin with other agents, such as lenalidomide, NVP-BEZ235 (PI3K inhibitor), and bortezomib, have been evaluated in non-Hodgkin lymphoma B-cell lines [13, 34, 35]. These studies can be considered as examples of new innovative attempts to identifying logical therapeutic combination.

Ibrutinib (PCI-32765) is an orally active inhibitor of BTK that binds Cysteine-481 on the kinase domain, leading to an irreversible inhibition at Tyr-223. Remarkable progress has been made in the development of ibrutinib in recent years, and the drug demonstrated considerable efficacy in a variety of B-cell malignancies. In ABC and GCB DLBCL, differences in activation of signaling pathways translate to differences in response to BTK inhibition, which have largely been confirmed in a Phase II trial of ibrutinib in relapsed DLBCL patients. Results from this trial revealed an overall response rate (ORR) of 37% (14/38) of patients with ABC-DLBCL, but only 5% (1/20) of patients with GCB-DLBCL [2]. Furthermore, ABC-DLBCL patients harboring CD79A/B^mut^, CARD11^mut^, TNFAIP3^mut^, or MYD88^mut^ showed primary resistance to ibrutinib [2, 6, 36]. On the other hand, as a result of activating mutations in BTK or PLCγ2, a subset of patients with an initially response to ibrutinib eventually relapse, underscoring the need for developing new target agents and combination treatments to improve the outcomes of such resistant patients [37]. Recent attention has been focused on potential drug combinations in DLBCL, particularly co-treatment with a BTK inhibitor and lenalidomide, bortezomib, PI3K inhibitor, or Pan-SRC kinase inhibitors in DLBCL [6, 29, 38–40]. Addition of ibrutinib to DLBCL cells treated with these agents resulted in synergistic cytotoxic effects on cells. There is also clinical data supporting the use of ibrutinib in combination with other agents, as combination therapy with rituximab and ofatumumab has been shown to be effective for the treatment of relapsed or refractory CLL/SLL [41]. Current on-going trials will further define the role of ibrutinib as upfront therapy and/or as a combination treatment in B-cell lymphoid malignancies. In the present study, analysis of the combination of PKCβ inhibitor enzastaurin and the BTK inhibitor ibrutinib has shown synergistic anti-tumor effects in DLBCL, thereby providing a rational basis for future preclinical/clinical investigations that may allow for the development of specific, well tolerated and efficient cancer therapeutics for relapsed or refractory DLBCL patients.

Another critical reason for supporting the combination treatment of enzastaurin with ibrutinib is that early studies have demonstrated the role of PKCβ in the negative regulation of BTK. Also, treatment with PKCβ inhibitors alter phosphorylation of BTK, leading to enhanced BTK signaling [18, 19]. Consistent with these previous works, our study also revealed that the expression of p-BTK was markedly increased by treatment of enzastaurin. Thus, PKCβ potently activates negative feedback signals of BTK, indicating that PKCβ inhibitors upregulate BTK’s activation thereby altering oncogenic signals downstream of BCR. Based on this mechanism, we investigated whether the combination of PKCβ inhibitor enzastaurin and BTK inhibitor ibrutinib had synergistic anti-tumor effects in DLBCL. Our findings revealed synergistic effects of these two agents on reduction of proliferation, promoting apoptosis, inducting G1 phase arrest, inhibition of cell invasion and migration, and downregulation activation of downstream signaling in GCB and ABC lymphoma cell lines.

Combination treatment of enzastaurin with ibrutinib has also been shown to trigger time-dependent inhibition of NOTCH1 mRNA expression, whereas treatment with either drug alone only slightly affected NOTCH1expression. The oncogenic role of NOTCH1 has been verified in a number of hematological diseases, including T-cell acute lymphoblastic leukemia, multiple myeloma, Hodgkin and anaplastic large cell lymphoma [23, 24, 26]. Many recent studies also have shown that a large number of DLBCL patients harbor NOTCH1 mutations and aberrations, validating the oncogenic role of NOTCH1 as the genetic drivers of DLBCL [42–44]. Moreover, NOTCH1 promotes the activation of the PI3K-AKT-mTOR and NF-κB signaling pathway, which plays a pivotal role in accelerating cell growth and promoting cell apoptosis not only in T-cell but also in B-cell neoplasms [24, 25]. In our works, treatment of DLBCL with a combination of enzastaurin and ibrutinib significantly reduced expression of NOTCH1, and shRNA mediated reduction in NOTCH1 expression dramatically inhibited DLBCL cell proliferation. These data indicated that downregulation of NOTCH1 could be a crucial biological mechanism by which the synergistic effect of co-treatment with enzastaurin and ibrutinib in suppressing cell growth. The precise mechanism in detail is likely to be a promising direction of further research.

## Conclusions

We have evaluated the combination of enzastaurin and ibrutinib in DLBCL in vitro and in vivo, demonstrating the co-treatment had synergistic anti-tumor effects in DLBCL, independent of molecular subtype. These results provide a sound foundation for further evaluation of an attractive therapeutic combination, suggesting that simultaneous inhibition of BTK and PKCβ may represent a novel, effective therapeutic approach for ABC and GCB DLBCL.

## Additional files

Additional file 1: **Table S1**. (DOC 64 kb)

Additional file 2: **Figures S1**-**S3**. (DOC 8170 kb)

## Abbreviations

ABC: activated B-cell-like
BCR: B-cell receptor
BTK: Bruton’s tyrosine kinase
CI: combination index
DFS: disease-free survival
DLBCL: diffuse large B cell lymphoma
GCB: germinal center B-cell like
GSK3β: glycogen synthase kinase 3β
IC50: 50% inhibitory concentration
KEGG: Kyoto Encyclopedia of Genes and Genomes
MM: multiple myeloma
NF-κB: nuclear factor κB
ORR: overall response rate
PARP: poly-ADP ribose polymerase
p-BTK: phosphorylated BTK
PKCβ: protein kinase C-β
p-PKCβ: phosphorylated PKCβ
SYK: spleen tyrosine kinase
WM: Waldenstrom’s macroglobulinemia
