# Supplementary material for: Combination of Enzastaurin and Ibrutinib synergistically induces anti-tumor effects in diffuse large B cell lymphoma
Source: J Exp Clin Cancer Res. 2019 Feb 18;38:86. doi: 10.1186/s13046-019-1076-4 (PMC6379963; doi:10.1186/s13046-019-1076-4)
Supplement: Supplementary file 1 — Table S1. (DOC 64 kb) [file 13046_2019_1076_MOESM1_ESM.doc]

**Supplemental Table**

**Table S1**. Information of antibodies applied in immunohistochemistry and western blot assays.

| Name(anti-) | Cat NO. | Concentration | Application |  |
| --- | --- | --- | --- | --- |
| PKCβ | 12919-1-AP | 1:3000 | Western blot | Proteintech |
| phospho–PKC(pan) (β II Ser660) | #9371 | 1:3000 | Western blot | CST |
| phospho–Ser661-PKCβ | #YP0432 | 1:50 | IHC | Immunoway |
| Phospho-Tyr223-BTK | #5082 | 1:2000 | Western blot | CST |
| Phospho-Tyr223/225-BTK | AF7354 | 1:100 | IHC | Affinity |
| BTK | #8547 | 1:3000 | Western blot | CST |
| PARP | 556362 | 1:3000 | Western blot | BD Bioscience |
| XIAP | #2045S | 1:3000 | Western blot | CST |
| Mcl-1 | #5453 | 1:3000 | Western blot | CST |
| Caspase 3 | #9662 | 1:3000 | Western blot | CST |
| Bcl-2 | #4223 | 1:3000 | Western blot | CST |
| CDK2 | #2546 | 1:3000 | Western blot | CST |
| CDK4 | #12790 | 1:3000 | Western blot | CST |
| CDK6 | #13331 | 1:3000 | Western blot | CST |
| Cyclin D1 | #2978S | 1:3000 | Western blot | CST |
| Phospho-Ser2448-mTOR | #2971 | 1:3000 | Western blot | CST |
| mTOR | #2972 | 1:3000 | Western blot | CST |
| Phospho-Tyr1217-PLCγ2 | #3871 | 1:3000 | Western blot | CST |
| PLCγ2 | #3872 | 1:3000 | Western blot | CST |
| Phospho-Ser9-GSK3β | #5558 | 1:3000 | Western blot | CST |
| GSK3β | #12456 | 1:3000 | Western blot | CST |
| Phospho-Thr202/Tyr204 -ERKk1/2 | #4370 | 1:3000 | Western blot | CST |
| ERK1/2 | #9102 | 1:3000 | Western blot | CST |
| NOTCH1 | #4380 | 1:2000 | Western blot | CST |
| Ki-67 | #ab16667 | 1:200 | IHC | Abcam, |
| β-actin | A5441 | 1:5000 | Western blot | Sigma-Aldrich |

IHC, immunohistochemistry; CST, Cell Signaling Technoligy, Danvers, MA, USA; Sigma-Aldrich, Darmstadt, Germany; BioRad, Hercules, CA, USA. Proteintech Group, Inc, Rosemont, IL, USA
