# Supplementary material for: Combination of Enzastaurin and Ibrutinib synergistically induces anti-tumor effects in diffuse large B cell lymphoma
Source: J Exp Clin Cancer Res. 2019 Feb 18;38:86. doi: 10.1186/s13046-019-1076-4 (PMC6379963; doi:10.1186/s13046-019-1076-4)
Supplement: Supplementary file 2 — Figures S1-S3. (DOC 8170 kb) [file 13046_2019_1076_MOESM2_ESM.doc]

**Supplemental Figure**


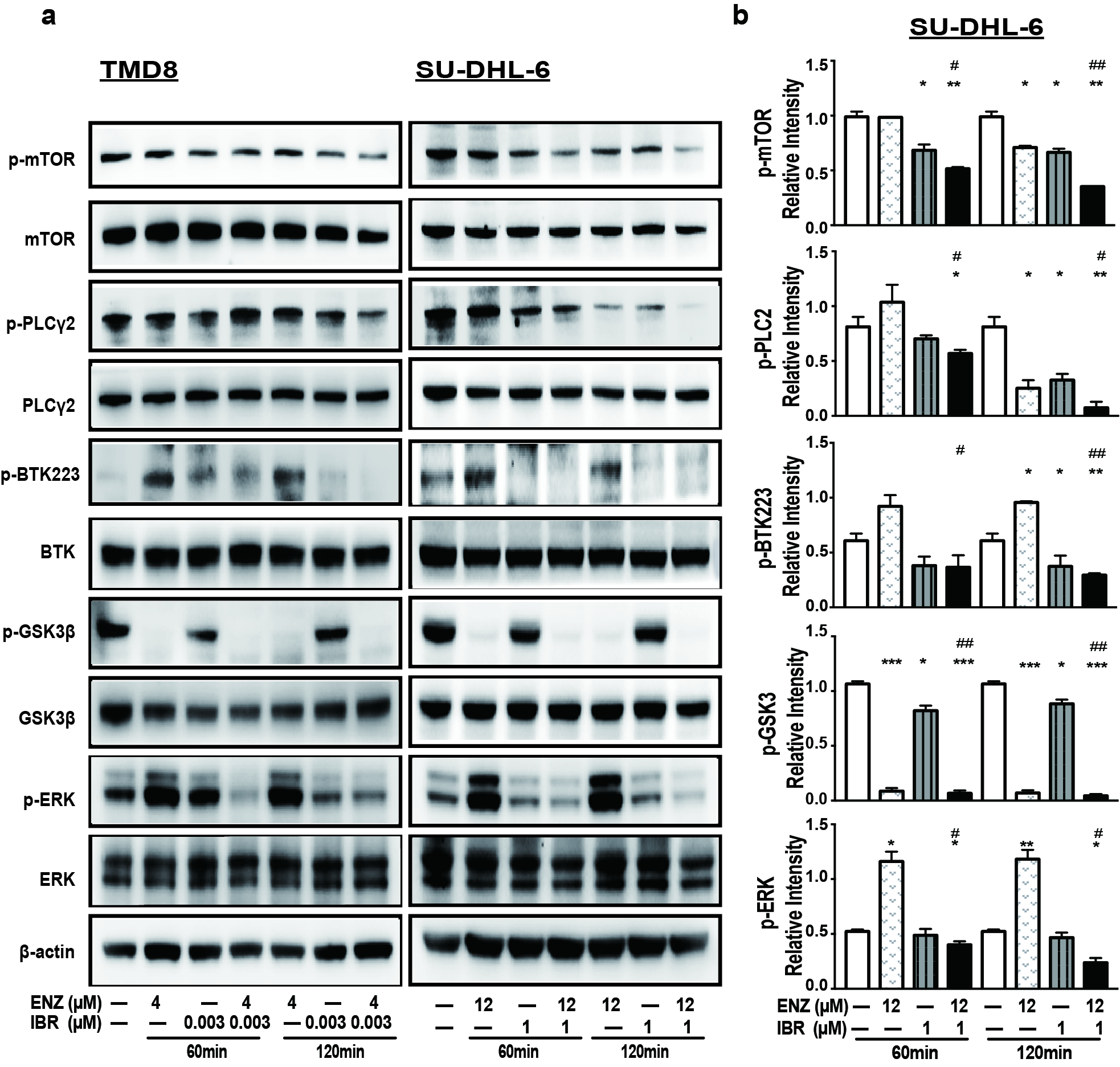


**Figure S1. Co-demonstration of enzastaurin and ibrutinib synergistically inhibit downstream signaling pathways.** (a) TMD-8 and SU-DHL-6 cells were treated with indicated concentration of enzastaurin alone, ibrutinib alone or enzastaurin plus ibrutinib for 60min and 120min, and cells harvested for western blot analysis. (b) The relative phosphorylation levels of signaling mediators were quantified by measuring the relative intensity of phosphorylated bands to the corresponding total bands, results are presented as mean ± SD of three scans. * p < 0.05, ** p < 0.01, *** p < 0.001 compared with control group; # p < 0.05, ## p < 0.01 compared with enzastaurin group.


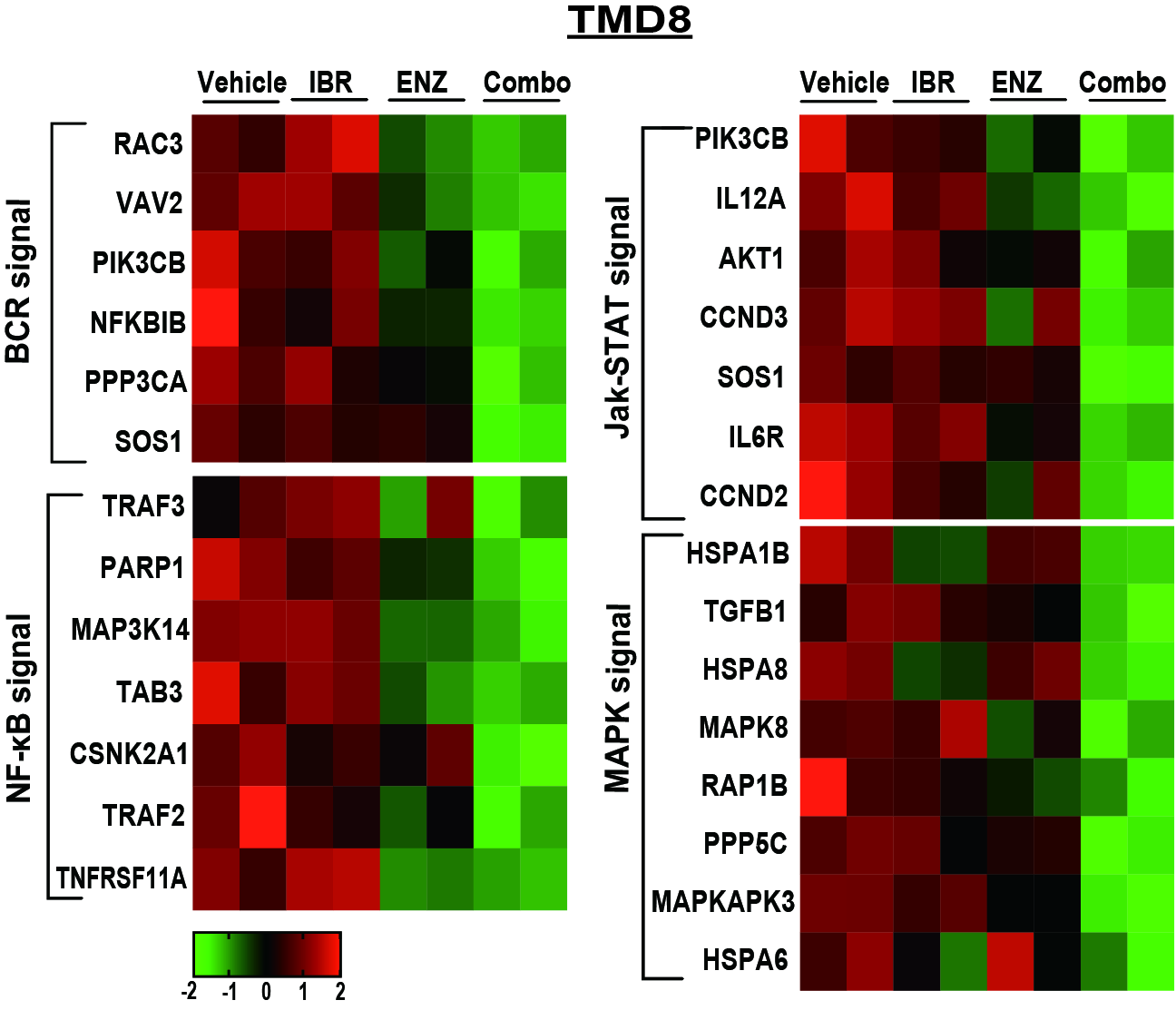


**Figure S2. Whole-transcriptome changes in TMD8 occur in response to the combination of enzastaurin and ibrutinib.** TMD-8 cells were exposed for 24 hours with 4 μM enzastaurin and/or 0.003 μM ibrutinib, RNA was collected for RNA sequencing. Significantly down-regulated genes from top ranked pathways (by KEGG) are represented in the heatmap.


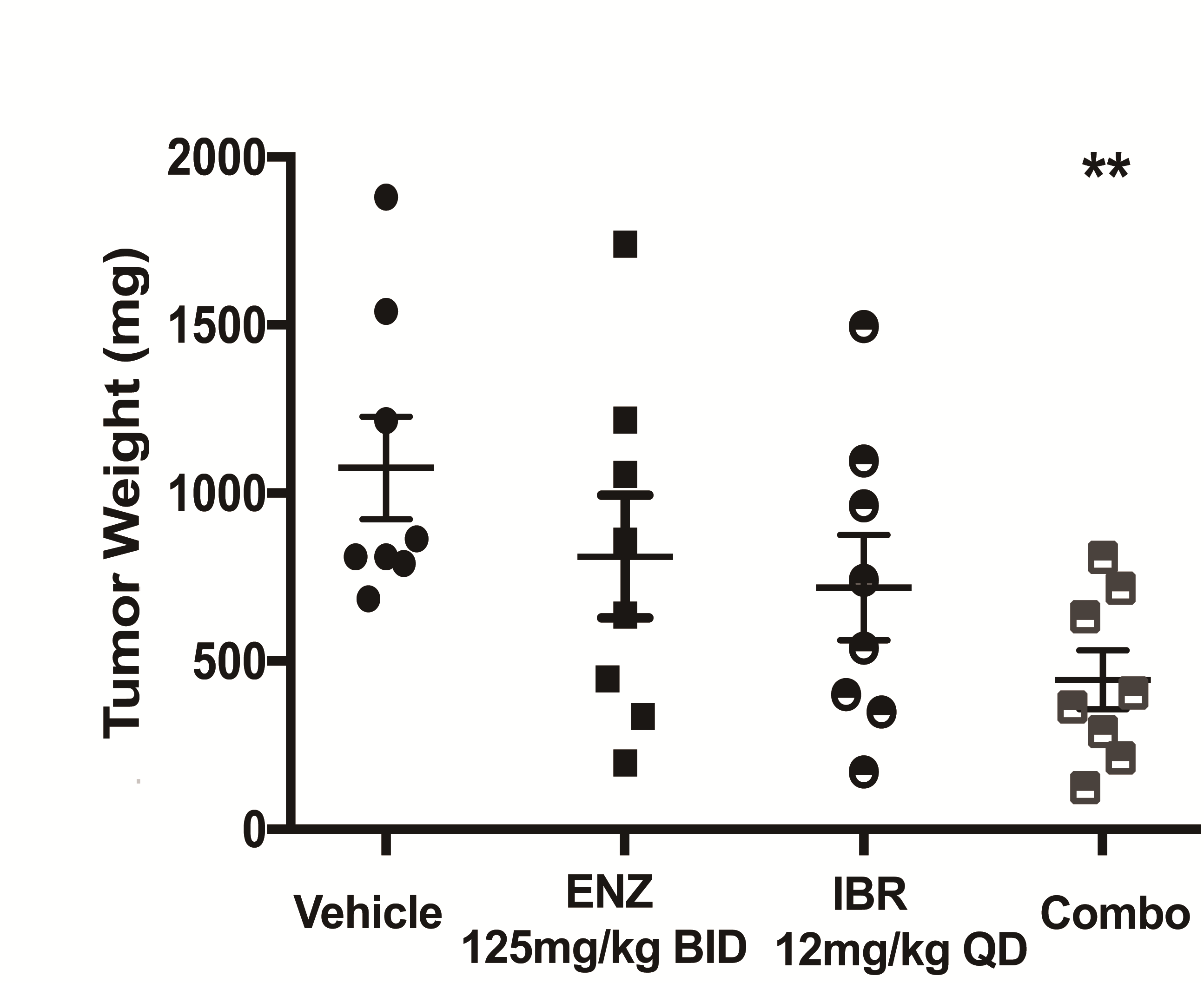


**Figure S3.** **Quantification of the tumor weight in the indicated mouse cohorts.** Results are expressed as mean ± SEM. ** p < 0.01 compared with vehicle group.
